# Glomerular Basement Membrane Thickness and IgA Nephropathy: A New Wrinkle?

**DOI:** 10.34067/KID.0000001255

**Published:** 2026-06-25

**Authors:** Sudhir Perincheri

**Affiliations:** Yale University School of Medicine, New Haven, Connecticut

**Keywords:** glomerular diseases, GN, IgA nephropathy, pathology

IgA nephropathy (IgAN) is among most common causes of GN worldwide. The clinical presentation can vary widely from asymptomatic hematuria to rapidly progressive GN. It presents with a broad variety of histologic changes by light microscopy and is characterized by dominant or codominant IgA deposits. The clinical course of IgAN can also vary widely from indolent to rapid progression to ESKD. In addition, there is also documented geographic variability in the clinical presentation and disease prognosis of IgAN.^1,2^

The Oxford classification system is a widely adopted, evidence-based pathologic classification scheme of IgAN that has shown variable correlation with long-term outcomes in both retrospective and prospective datasets.^3,4^ While ultrastructural changes have been documented in IgAN,^5^ they do not currently factor in the Oxford classification and their significance with regard to disease behavior remains unclear. In this issue, Zhengying *et al.* evaluated the significance ultrastructural abnormalities in a retrospective longitudinal cohort of 1006 patients with biopsy-proven IgAN with long-term follow-up and arrive at some interesting conclusions.^6^ They show that glomerular basement membrane (GBM) thickening as determined by the Nephrotic Syndrome Study Network metrics was correlated with mesangial hyperplasia and an independent risk factor for progression to ESKD in their IgAN cohort before and after adjusting for international risk-prediction tool in IgAN or the IgAN Oxford classification. This effect also seems to be independent of high arterial pressure or diabetes, two known causes of thickened GBMs, in their cohort.

These results could have important clinical implications if validated in other IgAN cohorts. Their observed correlation of GBM thickness and the M-score in their cohort is interesting given that M-score has been shown to be an independent predictor of outcome in independent studies.^7,8^ However, given the single-center retrospective nature of the study, the findings need to be replicated in other cohorts before they can become accepted and generalizable criteria for pathologic evaluation in IgAN. In addition, several sources of bias remain to be addressed. The reproducibility of Oxford mesangial hypercellularity, endocapillary hypercellularity, segmental glomerulosclerosis, tubular atrophy/interstitial fibrosis, and cellular/fibrocelluluar crescents scoring has been shown to be dependent on the expertise of the reviewing pathologist.^7^ The effect of treatment bias, *e.g*., with immunosuppression, if any, is not entirely clear. Added to this is the effect of known geographic variation in IgAN histopathology in various regions of the world. The measurement of GBM thickness is also fraught with sources of bias depending on the number and type of glomeruli imaged, the distribution of lesions and the methodology used. Validation in multi-institutional, multinational cohorts with centralized pathology review is necessary to address these concerns.

Renal pathology in an outlier in anatomic pathology in that it requires integration of data from histopathology, immunofluorescence microscopy, and electron microscopy for rendering a diagnosis. As is with the case with the Oxford classification, data from all the modalities may not feed into pathologic classification schema. The study by Zhengying *et al.* raises a broader question of whether the possibility exists to further refine classification and prognostic schema for various diseases in renal pathology by incorporating data from multiple modalities. The past decade has seen an explosion in digital and computational pathology techniques including multimodal artificial intelligence that can be used for objective and reproducible evaluation of tissue samples.^9^ These advances have led to regulatory approval of artificial intelligence tools in diagnostic pathology.^10^ Large-scale multi-institutional studies using these techniques in kidney diseases could result in better diagnostic and prognostic tools for optimal patient treatment. It could be argued that IgAN, with its diverse well-curated cohorts with follow-up data, is the ideal disease to test and showcase the value of such an approach in renal pathology.
